# Botanical Description of British Plants

**Published:** 1804-10-01

**Authors:** 


### *Botanical Description of British Plants.*

[Continued from pp. 220—232.]

40. BUNIUM. *B. flexuosum.*

*Ang.* Earth, kipper, pig, hawk or fur-nut; earth chesnut; lesser pig-nut.

*Gen. Desc.* Bloss. uniform: umbel crowded: Styles bent back, deciduous: seeds rather cylindrical, scored, thicker towards the end.

*Spec. Desc.* *Involucr.* from one to three leaves, deciduous. *Stem* leafless at the base, tapering downwards, zigzag. *Styles* permanent. *Root* tuberous, *Stem* smooth, scored, but little branched. Leaves doubly winged; segm. slender, tapering. Involucr. generally wanting. *Umbel* eight to twelve spokes. Umbellule sixteen sp. *Styles* at first close, afterwards straddling, never bent back. *Flowers* white. *Meadows, pastures, woods.* BLOSS. May, June.

*Use.* These roots eaten either raw, or boiled, or roasted, are scarcely inferior to chesnuts, and would be an agreeable addition to our winter deserts.

41. CONIUM. *C. maculatum. Cicuta major. Cicutaria vulgaris.*

*Ang.* Hemlock, common hemlock. Kex.

*Gen. Desc.* Invocellum going half-way round, of about three leaves. Fruit egg-shaped, bulging, ribs compressed, waved before the fruit is ripe.

*Spes. Desc.* *Seeds* without prickles. *Stem* branched, smooth, shining, spotted and streaked with blackish purple. *Involucel.* one leaf, div. into three and four; *segm.* at the edges white and membraneous. *Lower leaves* dark green and shining. *Outer petals* largest. *Flowers* white. *Hedges, orchards, dunghills, rubbish, and cultivated ground.* BLOSS. June, July.

*Use.* This plant has a peculiar faint fetid smell, and a slight aromatic and somewhat nauseous taste. That the plant is poisonous there can be no doubt, and numerous instances are recorded by various authors of its deleterious effects; but it seems probable, from some circumstances, that it is less powerfully so than was formerly imagined. That the root does not possess any noxious power whatever, several recent intances of its being repeatedly and largely eaten with impunity, have unequivocally been shewn. *Phil. Trans.* xix. p. 634. *Curtis Hor. Lond. Murray Ap. Med.* v. 1, p. 216. And Mr. Lane informed Mr. Curtis, that that from his own experience he was of opinion "the roots might be cultivated in gardens, and either eaten raw like celery, or boiled as parsnips and carrots." Vinegar has been found the most useful in obviating the effects of the poison of hemlock, and by macerating or boiling the plant in vinegar it becomes totally inert.—*Lindenstolpe de venenis.* For the principal symptoms produced by immoderate doses of hemlock, see *Haller, Murray, &c.*—This was generally employed by the Greek and Arabian physicians as an external remedy for tumours, ulcers, and cutaneous eruptions; it was also thought to have the peculiar power "frangere stimulum venereum," et "incrementa mammarum et testium cohibere:" which seems the more remarkable, as Stoerck, Bergius, and others, recommend its internal use in complaints of a contrary nature, and adduce proofs of its aphrodisiacal powers. *Berg. Mat. Med.* p. 195. Baron Stoerck first brought it into repute as a medicine of extraordinary efficacy, and Bergius considers its *virtus* to be *narcotica, resolvens, suppurationem promovens diuretica,* and recommends its use in various disorders; others, however, have condemned it, and the value of this medicine seems still to be undetermined. There are no testimonies in this country, like the facts adduced by Stoerck, of inveterate schirruses, cancers, ulcers, &c. hitherto deemed irremediable, having been cured by it; but it has been found that several disorders, which had resisted other medicines, have yielded to hemlock, and that some, if not cancerous, at least of that tendency, have been relieved by it. In chronic rheumatisms, glandular swellings, &c. &c. and in the chin-cough, it is now generally employed. Externally, the leaves of hemlock have been variously applied with advantage to ulcers, indurated tumours, and gangrenes.—*Woodville.* The difficulty of preparing the extract, and its great uncertainty, render the use of it dangerous and doubtful. Dr. Withering says, that for some years he has laid it aside, and prescribed only the powder of the dried leaves, for which he recommends the following method of preparation: Let the leaves be gathered about the end of June, when the plant is in flower. Pick off the little leaves and throw away the leaf-stalks. Dry these selected little-leaves in a hot sun, or on a tin dripping-pan, or a pewter dish, before the fire. Preserve them in bags made of strong brown paper, or powder them, and keep the powder in glass vials in a drawer, or something that will exclude the light; for the light soon dissipates the fine green colour, and with its its colour the medicine loses its efficacy. From fifteen to twenty-five grains of this powder may be taken twice or thrice a day. "I have found it peculiarly useful in *chronic rheumatisms,* and also in many of those diseases that are usually supposed to arise from acrimony. The nature of this book does not allow of minute details of the virtue of plants, but I can assure the medical practitioner that it is well worth his attention.—*Withering, l. c.* Mrs. Y. in Ireland, cured a poor woman of a cancer in the breast by hemlock pills taken inwardly, with stupes of the same plant.—*H. B.* Baron Stoerck used an *extract from the fresh root in spring,* in cancerous and scrophulous complaints.—*Lightfoot.*

42. Peucedanum. *P. officinale.*

*Ang.* Sulphur-wort. Hog's fennel. Harestrong.

*Gen. Desc.* Involucr. very short. Fruit, eliptical, slightly ridged, compressed, bordered.

*Spec. Desc. Leaves,* five times divided into three, thread strap shaped. *Petals,* yellowish. *Salt marshes.* Bloss. June, July.

*Use.* The roots have a strong fetid smell, and an acrid, bitterish, unctuous taste. When wounded in the spring, they yield a considerable quantity of yellow juice, which dries into a gummy resin, and retains the strong scent of the root. Its virtues have not yet been ascertained with precision.—*Withering.*

43. Crithmum. *C. maritimum. C. siculum.*

*Ang.* Rock samphire.

*Gen. Desc.* Florets equal. Fruit oval, compressed.

*Spec. Desc. Leafits* strap spear-shaped, fleshy. *Flowers,* white. *Sea coast.* Bloss. August.

*Use.* On the sea coast it is gathered for sale, being much used as a pickle: the poor people there eat it also as a pot-herb. Cows and sheep feed eagerly, and are said to grow fat upon it.—*Penn.*

44. Heracleum. *H. sphondyllium.*

*Ang.* Cow parsnip. Madnep. Hog-weed. Parsnip hog-weed.

*Gen. Desc.* Involucr. shedding. Bloss. irreg. Petals bent inwards, notched. Seeds compressed, leaf like, smooth, encompassed by a membranaceous border.

*Spec. Desc. Leafits,* wing-cleft, even. *Flowers,* radiated. *Leaf stalks,* at the base like a bag, scored, membranaceous, woolly at the edges. *Stem-leaves,* winged, hairy; *leafits,* three pair, jagged, indented; odd one, three-cleft. *Outer*

florets florets radiated, the *central* nearly equal. *Flowers* white. *Seeds* with three ridges on each side. *Hedges, meadows, pastures.* Bloss. July.

*Use.* In Poland and Lithuania the poor prepare a liquor from the leaves and seeds of this plant, which, after undergoing a fermentation, is used as a beverage like ale. The stalks, peeled, are eaten by the Kamschatdales. The Russians prepare from this plant an eatable, which they esteem a great delicacy: they pick off the leaf-stalks of the root-leaves, peel them, and hang them in the sun to dry a little; they then tie them in little bundles, and hang them up again till they become yellow; in this state they are put into bags, and a mealy substance, like sugar, forms upon the surface of them; this is carefully shaken off, and served as a treat to the guests. They likewise distill an ardent spirit from it.—*Gmelin.* The peelings of the stalks are acrid. The leaves are a favourite food of rabbits, hogs, and asses: cows, goats, and sheep eat them; horses are not fond of them.—*Withering.* This plant has been found useful in *epilepsy.*—*H. B.*

45. Ligusticum. *L. Scoticum.*

*Ang.* Scottish lovage. Sea parsley.

*Gen. Desc. Bloss.* equal. Petals rolled inwards, entire. Fruit oblong, tapering at each end, five ridges on each side.

*Spec. Desc.* Leaves doubly three-fold, glossy underneath. *Little leaves* oblong, wedge shaped, entire below, above serrated irregularly. *Rocks by the sea side.* Bloss. July.

*Use.* The root of this plant is reckoned a good carminative; an infusion of the leaves is said to be a good *purge for calves.* It is much valued in the isle of Sky, and is besides used as food, eaten either as sallad, or boiled as greens.—*Pennant.* Horses, sheep, and goats eat it: cows refuse it.—*Withering.*

46. Angelica. *A. archangelica. A. sativa.*

*Ang.* Garden angelica.

*Gen. Desc.* Bloss. equal, pet. bent inwards: styles reflected; fruit roundish.

*Spec. Desc. Leaves* winged; *leafits* unequally serrated, odd one three-lobed; the serratures broad and of a lopped appearance at the base: Involucell. sometimes longer than umbellule. *Broadmoore, near Birmingham.* Bloss. Sept.

*Use.* The medical character of angelica has made it an object of cultivation to the English gardener for more than two centuries. The stalk, leaves, seeds, but more particularly larly the root, have a fragrant agreeable smell and a bitterish pungent taste: on being chewed they are at first sweet, afterwards acrid, and leave a glowing heat in the mouth. " The fresh root, wounded early in spring, yields from the inner part of the bark an unctuous yellowish odorous juice, which gently exsiccated retains its fragrance, and proves an elegant aromatic gummy resin."—*Lewis.* Linnæus says, that the Laplanders entertain a very high opinion of the utility of this plant, as food and medicine, which is natural, as few aromatic plants inhabit the polar regions. Bergius thus enumerates its virtues: *Alexiteria, stomachica, sudorifera, carminativa;* and it has been recommended in female diseases; yet though it must be allowed to possess aromatic, and what are called carminative powers, it is in these qualities surpassed by other simples, and therefore seldom employed in the present practice.—*Woodville.* It is commonly used for a confection or sweetmeat; and employed in some distilled waters.

47. Angelica. *A. sylvestris.*

*Ang.* Wild angelica.

*Gen. Desc. As above.*

*Spec. Desc. Leafits* equal, egg-spear shaped, serrated finely and regularly. *Spokes* to forty; *Fruit stalks* to eighty. *Involucr.* generally 0. Involucel. permanent. *Bloss.* white, tinged with red. *Seeds,* border membranaceous, three ridges on the outer side. *Marshy woods and hedges.* Bloss. June, July.

*Use.* It is warm, acrid, bitter, and aromatic, possessing all the qualities of that cultivated in our gardens *(see Prec. Art.);* but as the latter possesses these properties in a higher degree, this has been long neglected. Cows, goats, and swine eat it; horses refuse it.—*Withering.*

48 Sium. *S. nodiflorum.*

*Ang.* Creeping water parsnip, or skerret.

*Gen. Desc. Involucr.* many leaved. Petals heart-shaped. Styles bent back; fruit roundish.

*Spec. Desc. Leaves* winged; *leafits,* tooth-serrated; *umbels* lateral, opposite the leaves. *Flowers,* white. *Stem* and *branches,* trailing or floating on the water. Involucr. deciduous. *Rivers and ditches.* Bloss. July, Aug.

*Use.* The efficacy of this plant is thus attested by Dr. Withering: " A young lady, six years old, was cured of an obstinate cutaneous disease by taking three large spoonfuls of the juice twice a day." Dr. W. has repeatedly given to adults three or four ounces every morning in similar similar complaints with the greatest advantage: it is not nauseous, he adds, and children take it readily if mixed with milk. In the doses of it given by him, it affected not either the stomach, the bowels, or the head.—*Withering.* It has lately been admitted into the Mat. Med. of the London College, in the character of an *antiscorbutic,* or rather as a corrector of acrid humours, especially when manifested by cutaneous eruptions, and tumours in the lymphatic system, on the testimony of Beiric and Ray.—*Woodville.*

49. Œnanthe. *Œ. crocata. Filipendula cicutæ facie. Ang.* Hemlock-dropwort. Dead tongue.

*Gen. Desc.* Florets of different shapes, the central fl. sitting, barren. Fruit with a cork-like coat, oblong, scored; crowned by permanent styles and calyx.

*Spec. Desc. Leaves,* many-cleft, blunt, nearly equal, some winged, some doubly winged. *Little leaves,* wedge shaped, smooth, streaked, jagged. *Petals* white, acute, bent inwards. *Involucr.* wanting, or of five strap-shaped leafits, readily falling off. *Umbellule,* nearly globular. *Gen. Bloss.* not very unequal. *Watery places, banks of rivers and ditches.* Bloss. June, July.

*Use.* An infusion of the leaves, or three tea spoonsfull of the juice of the root taken every morning, effected a cure in a very obstinate *cutaneous disease;* but it was not without occasioning very great disturbances in the constitution. *See Dr. Pulteney's letter, Phil. Trans. lxii. p.* 469. The whole of this plant is *poisonous;* and Dr. Pulteney remarks, that of all the vegetable poisons produced in Great Britain, the root of this plant is the most virulent. Many instances of its fatal effects are recorded; for which see *Phil. Tr. ib. and vol. i. p.* 856. *Gent. Mag. July,* 1747, *Mar.* 1755, *Sept.* 1758. It is made use of by the country people in Westmoreland as an application, in the form of a poultice, to the ulcer, which forms in the fore part of the cleft of the hoof in horned cattle, and is called *the foul.*—*Withering.* This plant has been sometimes mistaken for celery, to which it bears some resemblance; but it is a *terrible poison,* and even the smell of it is deleterious: a vomit is the best remedy for those who have eaten of it. A spoonful of the juice given to a dog, rendered him sick and stupid: a goat eat it with impunity.—*Lightfoot.* For instances of its deleterious effects, see Dr. Woodville, Sir William Watson, Phil. Trans. vol. xliv. Allen Synop. Med. &c.

50. Phellandrium. *P. aquaticum. Cicutaria palustris. Fœniculum aquaticum.*

*Ang.* Water hemlock. Horsebane.

*Gen. Desc.* Central florets smallest; fruit egg shaped, smooth; crowned with the pistil and the calyx.

*Spec. Desc.* Ramifications of the leaves straddling. *Stem* very thick. *Leaves,* under water long, hair like. *Petals,* white. *Rivers, ditches, pools.* Bloss. June, July.

*Use.* The medicinal quality of this plant now rests chiefly on the testimonies of Erstingius and Lange, by whom various cases of its successful use are published, especially in *wounds* and inveterate *ulcers* of different kinds, and even in *cancers;* also in *phthisis pulmonalis, asthma, dyspepsia, intermittent fevers, &c.* About two scrs. of the seed, two or three times a day, was the ordinary dose given. Boerhaave speaks highly of its discutient power in all kinds of tumours. *Hist. Plant. Hort. Ludg. Bat.* 1. *p* 94. The medicinal qualities of these seeds are not satisfactorily ascertained, but they appear to be well deserving of further investigation.—*Woodville.* The seeds are recommended in intermittents; and are said to be *diuretic, antiseptic,* and *expectorant:* dose one to three drachms daily.—*Dr. Lange.* The leaves are sometimes added to *discutient* cataplasms. It is commonly esteemed a fatal poison to horses, occasioning them to become paralytic; but this effect is owing to a certain insect *(the curculis paraplecticus)* which generally inhabits within the stems. The usual *antidote* is pig-dung. In winter, the roots and stem, dissected by the weather, afford a very curious skeleton or net-work. Horses, sheep, and goats eat it; swine are not fond of it; cows refuse it.—*Withering.*

51. Cicuta. *C. virosa. C. aquatica. Sium alterum.*

*Ang.* Long leaved water hemlock. Water cowbane.

*Gen. Desc.* Fruit nearly egg shaped, ribbed.

*Spec. Desc. Leaves,* winged; *leafits,* spear shaped, in threes, serratures white at the point. *Stem,* four feet high, reddish below. *Fruit-stalks,* sheathed at the base. *Umbel,* expanding, red at the base. *Styles,* upright, white, in the fruit straggling. *Fruit,* compressed, even, lopped. *Petals,* yellow green. *Pools.* Bloss. July, Aug.

*Use.* As an internal medicine this is universally superseded by the common hemlock; but externally employed in the way of poultice, it is said to afford relief in various fixed pains, especially those of the *rheumatic* and *arthritic* kind.

kind. In its dried state, Bergius tells us, it may be taken in considerable quantities without producing any bad effect; but the root, when fresh, is extremely deleterious. The symptoms produced upon some children, who ate of it for parsnip root, were intoxication, vertigo, great heat and pain in the stomach, convulsions, and even epilepsy, distortions of the eyes, vomiting or retching, discharge of blood from the ears, swelling of the abdomen, hiccup, spasms, &c. In a man, delirium, with constant heat of the stomach, and unextinguishable thirst, were of long continuance, and followed by an erysipelatous tumour in the neck. See *Eph. Nat. Cur. Cent.* 10. Obs. 58. p. 355. The timely administration of an emetic is the only remedy.—*Woodville.* It is one of the rankest of our vegetable poisons. Numerous instances are recorded of its fatality to the human species in a treatise upon it by *Wepfer* and by *Haller,* as well as in the *Phil. Trans. abr.* x. Early in the spring, when it grows in the water, cows often eat it, and are killed by it; but as the summer advances, its scent becomes stronger, and warns them carefully to avoid it. But, though it is a certain and fatal poison to horned cattle, goats devour it greedily and with impunity. Horses and sheep also eat it with safety.—*Withering.*

52. Æthusa. *Æ. cynapium.*

*Ang.* Fool's parsley, or cicely. Lesser hemlock.

*Gen. Desc.* Involucell. reaching half way round, three-leaved, bent downwards; fruit nearly globular, deeply furrowed.

*Spec. Desc. Leaves,* all alike, doubly winged, smooth, glossy dark green; *leafits* div. into segm. subdiv. into three or five. *Stem.* one and a half to two feet high, branched. *Umbel.* often eighteen sp. *Flowers,* whitish. *Corn fields, kitchen gardens.* Bloss. August, Sept.

*Use.* From the resemblance of this plant to common parsley, it has often been mistaken for the latter; and when eaten, it occasions sickness. It is noxious to geese. Horses, cows, sheep, goats, and swine eat it.—*Withering.*

53. Æthusa. *Æ. meum. Athamanta meum. Ligusticum meum. Seseli meum.*

*Ang.* Spignel. Spicknel. Men. Bald or bawd money. Spignel cicely.

*Gen. Desc. As above.*

*Spec. Desc. Leaves* divided into many bristle-shaped segments; *involucr.* 1; leaf, sometimes none; *fruit,* egg oblong long, tapering at each end. *Petals*, white. *Mountainous pastures.* Bloss. May.

*Use.* Linnæus says, that the radical fibres of this plant, form the basis of the calculus ægagropila, but though I have examined several of these balls, I never found it so. *Mr. Gough.* The roots and seeds are aromatic and acrid. They have been used as stomachics and carminatives; sometimes they are given to cure tertians; and there is no doubt but they will often answer as well as pepper, and other acrid aromatics.—*Withering.*

54. Coriandrum. *C. sativum. C. majus. C. vulgare.* *Ang.* Common coriander.

*Gen. Desc.* Bloss. radiated; petals bent inwards, notched at the end; involucr. one leaf; involucell. reaching half way round; fruit globular, smooth.

*Spec. Desc.* *Fruit* globular. Whole plant smooth. *Leaves* cut into very slender strap shaped segments. *Proper calyx* five leaves, permanent. *Styles* permanent, reflected. *Outer florets* of umbellules barren; petals larger, expanding, radiated; *central florets* fertile; *petals* equal, bent inwards. *Flowers* whitish. *Seeds* two, united, so as to form a globe. *Corn-fields, road-sides, dunghills.* Bloss. June, July.

*Use.* The leaves and every part of the plant, when fresh, have a very offensive odour, like bugs; but upon being dried, the seeds have a tolerably grateful smell, and their taste is moderately warm and slightly pungent. These seeds, it is asserted by Dioscorides, if taken in any considerable quantity, produce deleterious effects; and in some parts of Spain and of Egypt, where the fresh herb is eaten as a cordial, instances of fatuity, lethargy, &c. are observed to occur very frequently,—*Hoffman;* but these properties seem to have been unjustly ascribed to the coriander; and Dr. Withering says, that though they have been considered as suspicious, if not deleterious, he has known six drachms of them taken at once without any remarkable effect. These seeds, like those of most of the umbelliferous plants, possess a stomachic and carminative power; but they are principally of use, according to Dr. Cullen, "as correctors of the bitter infusion and the preparations of senna, nothing so powerfully covering its disagreable odor and taste, and it being equally efficacious in obviating the griping that senna is very ready to produce." *Woodville; Cullen Mat. Med. ii. p.* 153. The seeds incrusted with sugar are sold by confectioners, under the name of coriander comfits.

55. Scandix. *S. odorata.*

*Ang.* Sweet Cicely. Shepherd's needle. Great sweet chervil. Sweet fern.

*Gen. Desc.* Bloss. radiated; central florets frequently male; petals notched at the end; styles permanent; fruit awl-shaped.

*Spec. Desc.* *Seeds* furrowed, angular, longer than the umbellules, of a sweet and agreeable taste. *Leaves* trebly winged; *little leaves* with winged clefts; *segments* deeply and sharply serrated. *Flowers* white. *Whole plant* of an aromatic scent. *Orchards and waste places, but always near houses.* Bloss. June.

*Use.* The seeds of this plant are very commonly used in the north of England for polishing and perfuming oak floors and furniture.—*Woodward.*

56. Scandix. *S. cerefolium.*

*Ang.* Common chervil. Chervil shepherd's needle.

*Gen. Desc.* *As above.*

*Spec. Desc.* *Seeds* glossy, cylindrical, beaked. *Umbels* generally lateral, nearly sitting. *Leaves* exceedingly delicate. *Spokes* woolly, four, sometimes three or five; of umbellule ten and twelve. *Involucr.* leaf strap shaped. *Bloss.* white. *Hedges.* Bloss. May.

*Use.* This plant is slightly *aromatic* and *aperient.* It is frequently cultivated in our gardens as a pot herb, and is used in sallads. Cows are extremely fond of it; sheep and goats eat it; horses refuse it.—*Withering.*

57. Chærophyllum. *C. sylvestre. Myrrhis.*

*Ang.* Wild Cicely. Cow weed. Cow parsley. Cow weed chervil.

*Gen. Desc.* *Invollucell.* reflected, concave; petals heart shaped, bent inwards; fruit shining, generally smooth, oblong.

*Spec. Desc.* *Stem* smoothish, scored, swoln at the knots, woolly. *Central florets* of umbellules often barren. *Flowers* white. *Hedges, orchards, pastures.* Bloss. May, June.

*Use.* In some parts of the kingdom, in times of scarcity, this plant is eaten as a pot-herb.—*Curtis.* The roots have sometimes been eaten as parsneps, but they have been found poisonous. The umbels afford an indifferent yellow dye; the leaves and stem a beautiful green. Its presence indicates a fruitful soil. Neither horses, sheep, goats, nor swine are fond of it; rabbits eat it greedily; and cows are very very fond of it, so much so, that when, as often happens about Dudley, a pasture is over-run with it, cows are turned in to eat it up.—*Withering.*

58. IMPERATORIA. *I. ostruthium. Astrantia vulgaris. Magistrantia.*

*Ang.* Common masterwort.

*Gen. Desc.* Petals bent inwards, notched at the end; seeds compressed with a broad membranaceous border, and three ridges on the back.

*Spec. Desc.* But one species has been described. *Banks of the Clyde.* BLOSS. June.

*Use.* The root has a fragrant smell and a bitterish pungent taste, leaving for some time a glowing warmth in the mouth. This plant was formerly, as its name imports, thought to be of singular efficacy, and was preferred to most of the other aromatics, for its alexipharmic and sudorific powers. In some diseases it was employed with so much success as to be distinguished by the name of "divinum remedium."—*Hoffman.* At present, however, this root being considered merely as an aromatic, is superseded by many of that class, of superior character; half a drachm of the root in substance, and one drachm of it in infusion, is the dose directed.—*Woodville.* The root is warm and aromatic; it is a *sudorific*, a *diuretic*, and a *sialogogue;* recommended in *dropsy* and *debilities* of the stomach and bowels; and an infusion of it in wine, is said to have cured *quartans*, which had resisted the bark.—*Dr. Stokes.* When chewed it excites a copious flow of saliva, exciting a warm and not disagreeable sensation in the gums, and frequently curing the rheumatic *tooth-ache.*—*Withering.* It should be dug up in winter, and a strong infusion made in wine.—*Lightfoot.*

59. PASTINACA. *P. sativa.*

*Ang.* Wild parsnep. *The* garden parsnep *is a variety, altered only by culture.*

*Gen. Desc.* Petals rolled inwards, entire; seeds eliptical, compressed, leaf-like, smooth; border thin, narrow.

*Spec. Desc. Leaves* simply winged. *Stem* three or four feet high, membranaceous at the corners. *Involucr.* O. *Umbel spokes* six to twelve; *Umbellule spokes* short, numerous. *Involucellum* sometimes one leaf. *Flowers* yellow. *Borders of ploughed fields, in lime-stone.* BLOSS. June, July.

*Use.* The roots of this plant are sweeter than carrots, and, when improved by garden culture, are an excellent vegetable for the table, and much used by those who ab-

 stain stain from animal food in Lent; they are highly nutritious. In the north of Ireland they are brewed instead of malt, with hops, and fermented with yeast; the liquor thus obtained is agreeable. The seeds contain an essential oil; and will often cure intermittent fevers. Hogs are fond of the roots, and quickly grow fat upon them.—*Withering.*

[To be continued.]

